# The Effect of Whitening Creams Usage on the Adrenal Gland 

**DOI:** 10.12688/f1000research.173725.2

**Published:** 2026-07-31

**Authors:** Fadheelah Salman Azeez, Noor Faisal Noaman, Ahmed Neema AL-Mussawy

**Affiliations:** 1 Polytechnic College Hawija, Northern Technical University, Hawija, Iraq; 2 Department of Physiology, Biochemistry and Pharmacology, College of Veterinary Medicine, University of Kirkuk, Kirkuk, Iraq; 3 Department of Biology, College of Science, Kerbala University, Kerbala, Iraq

**Keywords:** Skin-Whitening Products, Cortisol, Adrenal Suppression, BMI

## Abstract

**Background:**

This cross-sectional study assessed the impact of long-term use of skin-whitening products containing corticosteroids on the function of the adrenal glands.

**Objectives:**

Evaluate morning serum cortisol levels among habitual users of corticosteroid- containing skin whitening creams compared with non-users.

**Methods:**

This research, executed at the Hawija Technical Institute from January to April 2025, included 57 participants aged 20 to 40. Before enrolling, all participants gave their written consent. Individuals with chronic endocrine disorders or those receiving systemic corticosteroids were excluded. They were split into two groups: 38 regular (14 male and 24 female) users of skin-whitening creams (≥3 months) as the experimental group and 19 (7 male and 12 female) non-users as the control group. The Mindray CL-900i, a fully automated chemiluminescence immunoassay analyzer, was used to measure the levels of cortisol in the serum.

**Results:**

In comparison to controls (7.85 ± 3.54 μg/dL; p = 0.0004), users had a significantly lower mean cortisol level (4.25 ± 2.23 μg/dL), with 42% of users exhibiting morning serum cortisol levels <5 μg/dL, which may suggest reduced hypothalamic-pituitary-adrenal (HPA) axis activity. There were no discernible variations in BMI between the groups or between male and female users. These findings suggest an association between prolonged exposure to corticosteroid-containing skin whitening creams and reduced morning serum cortisol levels, consistent with possible suppression of the HPA-axis activity.

**Conclusions:**

Prolonged use of corticosteroid-containing whitening creams was associated with lower morning serum cortisol levels, suggesting reduced HPA-axis activity. Further studies are needed to confirm HPA-axis suppression.

## Introduction

In many parts of the world, especially in Asia, Africa, and the Middle East, where lighter skin is frequently linked to social standing and beauty, skin-whitening creams have grown in popularity. ^1, 2^ Corticosteroids, hydroquinone, and mercury compounds are common active ingredients in these products. When used excessively or without medical supervision, these substances can cause serious systemic side effects. ^3, 4^

The effects of long-term use of corticosteroid-containing whitening creams on the endocrine system, especially the adrenal glands, are one of the main causes for concern. ^5, 6^ Local research has similarly observed endocrine dysregulation and HPA axis activation in patients with thyroid disorders in Kirkuk. ^7^

Recent studies have demonstrated that the skin is not only a passive barrier for topical drug absorption but also functions as an active neuroendocrine organ. Human skin possesses a functional cutaneous hypothalamic–pituitary–adrenal (HPA) axis equivalent capable of producing corticotropin-releasing hormone (CRH), proopiomelanocortin (POMC)-derived peptides, adrenocorticotropic hormone (ACTH), and cortisol. This local neuroendocrine system plays an important role in maintaining skin homeostasis and coordinating responses to environmental and physiological stressors. Consequently, prolonged exposure to topical corticosteroids may influence both local cutaneous endocrine regulation and the systemic HPA axis through percutaneous absorption, providing a biological basis for their potential endocrine effects. ^8, 9^

The production of essential hormones like cortisol, aldosterone, and androgens is carried out by the adrenal cortex. ^10– 12^ The hypothalamic-pituitary-adrenal (HPA) axis can be suppressed by exogenous corticosteroids that are absorbed through the skin, resulting in Cushing’s syndrome or adrenal insufficiency. ^13, 14^

Long-term topical steroid use has been linked to an increasing number of patients experiencing metabolic problems and adrenal suppression, according to recent clinical studies and case reports. ^15^ Many over-the-counter whitening products are still unregulated, despite regulatory efforts, which puts the public’s health at serious risk. ^16^

With an emphasis on the mechanisms of hormonal disruption and possible clinical outcomes, this study attempts to investigate the physiological effects of skin-whitening creams on adrenal gland function. ^17^

The recovery of the hypothalamic–pituitary–adrenal (HPA) axis following corticosteroid administration is contingent upon the dosage, potency, and duration of use. ^18^

• ACTH levels start to rise again in 1 to 4 weeks.
• Partial recovery can happen in 1 to 3 months.
• Full recovery can take 3 to 12 months, and in long-term high-potency use, suppression can last more than a year. To avoid an adrenal crisis, it is important to gradually taper off. ^19^

Difficulties Associated with the Use of Corticosteroids or Skin-Whitening Creams. Using skin-whitening creams that contain corticosteroids can be very bad for your health. They might make your skin look lighter for a short time, but using them for a long time or without supervision can cause skin thinning, acne, striae, telangiectasia, and HPA axis suppression. ^20^ A lot of people use these products without talking to a doctor first, usually because of cultural pressures or ads that aren’t true. Stopping them suddenly may cause rebound dermatitis or withdrawal symptoms. Overall, the misuse of these creams is a big medical and regulatory problem that needs more attention and control. ^21^

The primary objective of our study was to evaluate morning serum cortisol levels among habitual users of corticosteroid-containing skin-whitening creams compared with non-users.

## Materials and method

### Study design

This cross-sectional study was conducted from January to April of 2025. All the Fifty-seven participants both male and female, ages of 20 – 40 years. Everyone who took part gave their written consent before doing so. Individuals with chronic endocrine disorders or those undergoing systemic corticosteroid therapy were excluded. They were divided into two groups: an experimental group and a control group.

### Sample size justification

The sample size of 57 participants was established to represent the maximum number of eligible participants attainable during the study period, informed by prior analogous studies, thereby ensuring adequate statistical power (>80%) to identify variations in serum cortisol levels between users and controls.

### Participants

Group 1 (Experimental group) included 38 participants who reported using skin-whitening creams regularly for at least three consecutive months. The products were taken from local beauty salons as cosmetic mixtures without medical supervision. No laboratory chemical analysis was performed to identify the corticosteroid content, concentration, or potency of the products used by the participants. The experimental group was split up into 24 females and 14 males. Group 2 (Control group) consisted of 19 participants (7 male and 12 female) who reported never used any topical skin-whitening or corticosteroid-containing products. The unequal group ratio (1:2, 19 controls and 38 users) is based on the number of people who could participate and the fact that many people use skin-whitening cream regularly. It still gives us enough statistical power to make comparisons. Structured interviews were administered to each participant to gather data on cream usage and demographic attributes. The length of time people used the cream varied from 3 to 12 months. Prior to sample collection written informed consent was obtained from all participants involved in this study.

### Sample collection

The sample size was established based on feasibility and prior analogous studies. Venipuncture was used to take blood samples (5 mL) from all participants between 8:00 and 10:00 in the morning. We let the samples clot at room temperature and then used a standard centrifuge to separate the serum.

### Hormone measurement

The Mindray CL-900i, a fully automated chemiluminescence immunoassay analyzer, was used to measure cortisol levels.

### Statistical analysis

SPSS was used for statistical analysis, and an independent t-test was used to compare the experimental and control group’ cortisol levels; a p-value of less than 0.05 was deemed statistically significant.

## Results

This study concludes 57 participants (34 female and 23 male), that divided into experimental group 38 (24 female and 14 male) and control group 19 (12 female and 7 male), the two groups age were 22.92 ± 3.26 and 21.21 ± 2.30 years old respectively. Table 1 show these informations:

**Table 1.** Distribution of the experimental and control groups according to their age and gender.

Group	Age average ± SD	Male	Female	Total
Experimental group	22.92 ± 3.26	14	24	38
Control group	21.21 ± 2.30	7	12	19
Total		21	36	57

**Figure 1.**
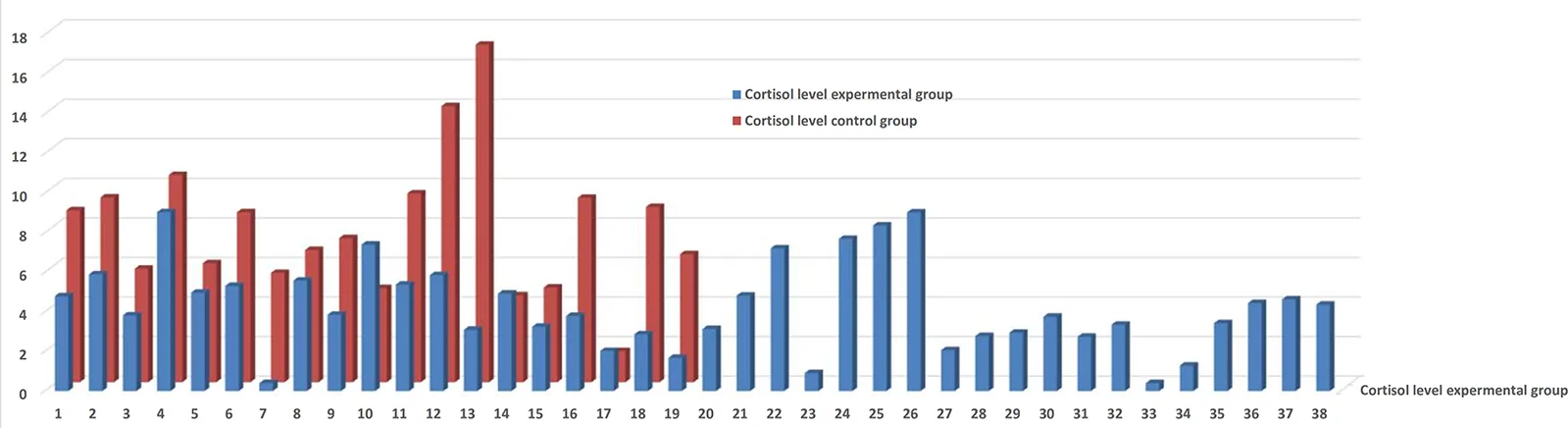
Mean cortisol level of experimental vs. control group.

The control group had a significantly higher mean cortisol level (7.85 ± 3.54 μg/dL) in comparison with the experimental group (4.25 ± 2.23 μg/dL) (normal range of morning cortisol 6-25 μg/dL). The experimental group had significantly lower morning serum cortisol levels than the control group (p = 0.0004), suggesting a possible reduction in hypothalamic-pituitary-adrenal (HPA) axis activity as shown in Table 2 and Figure 1.

**Table 2.** Comparison of cortisol value between experimental group and control group.

Group	Cortisol ± SD (μg/dL)	Min	Max	P-value
Experimental Group	4.25 ± 2.23	0.4	9.05	0.0004
Control Group	7.85 ± 3.54	1.58	17.6	

**Figure 2.**
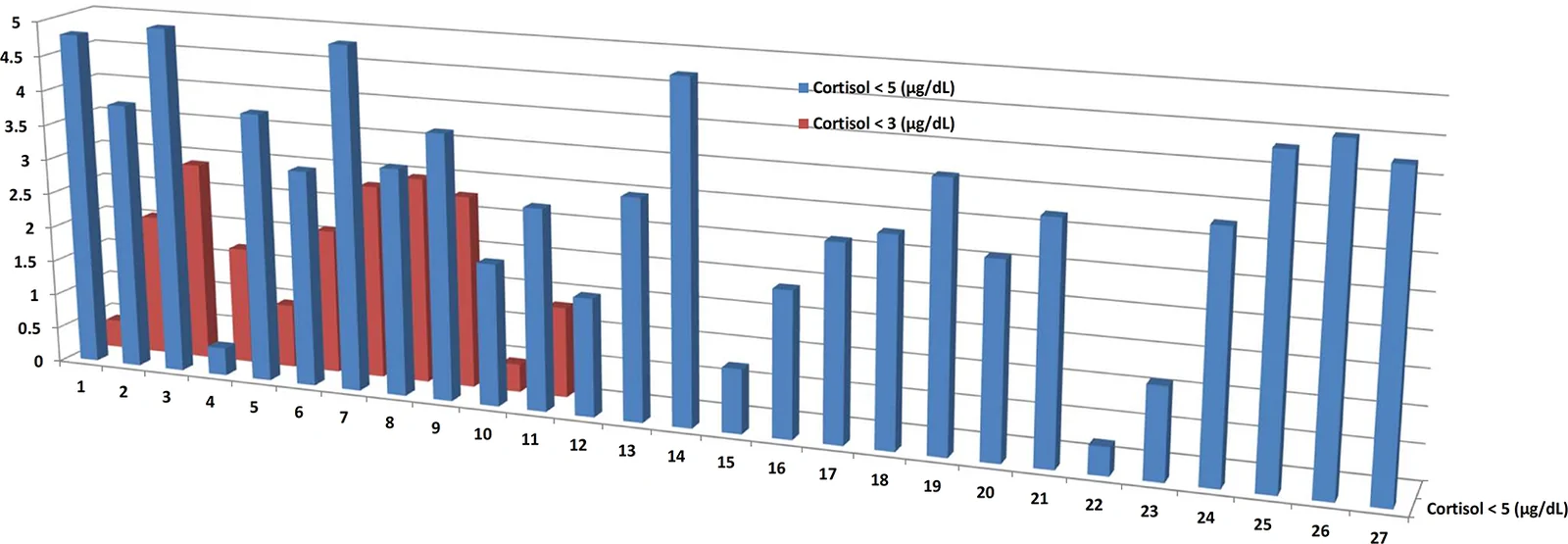
Cortisol level comparison in between users.

Morning serum cortisol levels below 5 μg/dL were observed in about 42% of users and below 3 μg/dL in 18% as shown in Figure 2.

The mean cortisol level in the experimental group (users) was 4.06 μg/dL (SD ± 1.80) for females and 4.57 μg/dL (SD ± 2.88) for males. There are 24 women and 14 men among the participants. Cortisol levels ranged from a minimum of 1.68 μg/dL for females and 0.40 μg/dL for males to a maximum of 9.05 μg/dL and 9.04 μg/dL, respectively. There is no statistically significant difference in cortisol levels between male and female participants, according to Table 3’s p-value of 0.563 from an independent t-test that was used to compare the two groups.

**Table 3.** Comparison of cortisol value between male and female users.

Gender	Mean ± SD	Min	Max	Count	p-value
Female	4.0625 ± 1.803083	1.68	9.05	24	0.562951
Male	4.565 ± 2.881396	0.4	9.04	14	

The mean BMI in the control group was 26.03 kg/m ^2^ (SD ± 3.17), while in the experimental group it was 25.15 kg/m ^2^ (SD ± 3.17) ( Table 4). Despite the control group’s marginally higher mean BMI, there was no statistically significant difference between the two groups (p = 0.330).

**Table 4.** Comparison of BMI between the experimental group and control group.

Group	Mean ± SD	Min	Max	Count	p-value
Control	26.02617 ± 3.172375	22.22222	34.60208	19	0.330056
Experimental	25.14606 ± 3.173055	16.66667	32.46191	38	

## Discussions

With mean values of 4.25 ± 2.23 μg/dL and 7.85 ± 3.54 μg/dL, respectively, the experimental group (users) had significantly lower serum cortisol levels than the control group (p = 0.0004). The significantly lower morning serum cortisol levels observed among users may suggest reduced HPA-axis activity following prolonged exposure to topical corticosteroids. Previous studies have similarly reported that prolonged topical corticosteroid use may affect HPA-axis function; however definitive confirmation of HPA-axis suppression requires additional endocrine evaluation, including ACTH measurement and dynamic testing. This finding is in line with earlier research that connected inappropriate or long-term topical corticosteroid use to adrenal suppression. ^22, 23^ According to dermatological and endocrinological literature, prolonged exposure to glucocorticoids, even through dermal absorption, can impair adrenal responsiveness. ^13, 24^ Several factors, including skin integrity, steroid potency, and the duration and frequency of use, influence percutaneous corticosteroid absorption, which may lead to significant changes in systemic steroid levels as demonstrated in recent pharmaceutical research. ^25^

In the present study 42% of users had morning serum cortisol levels below 5 μg/dL, while 18% had levels below 3 μg/dL. These findings are consistent with thresholds commonly used to identify individuals at risk of adrenal insufficiency in clinical practice. ^26, 27^

However, morning serum cortisol alone cannot establish HPA-axis suppression without confirmatory endocrine testing. ^28^

The analysis that gender-based exposed that the male participants of cortisol levels was (4.57 μg/dL) and that of female participants was (4.06 μg/dL) which did not differ statistically significantly (p = 0.563), demonstrating that the observed reduction in morning serum cortisol levels was not associated with sex. This finding is in consistence with previous research finding that the biological sex has less effect on the systemic effects of potent topical corticosteroids than steroid potency, application surface area, and treatment duration. ^29, 30^ The body mass index (BMI) of the control group average was higher rather at 26.03 kg/m ^2^, while the average BMI of experimental group was 25.15 kg/m ^2^. Though, the difference (p = 0.330) was not statistically significant. This suggests that BMI is improbable to have an effect on the detected cortisol variations. Epidemiological studies demonstrated similar results that have revealed that the inhibition of the HPA axis is not always associated with BMI in corticosteroid users. ^31^

Following the use of topical corticosteroids for long period, the HPA axis may require weeks to months for recovery, dependent on the potency, duration of use, and individual sensitivity. This reveals how essential it is to screen them and cease using them gradually to prevent adrenal crises. ^32^

### Clinical implications

This study highlights the clinical importance of evaluating adrenal function in individuals who regularly use corticosteroids containing skin-whitening products. Physicians should be aware that excessive absorption of topical corticosteroids may result in adrenal gland dysfunction. Regular cortisol testing and patient education regarding the safe use of topical corticosteroids are essential to help prevent adrenal complications, especially in populations where supervision of cosmetic steroid usage is prevalent. Health authorities should strengthen regulations and require clear product labeling to reduce misuse of these products.

To protect people and lower the risk of endocrine problems, the FDA, WHO, and other regulatory bodies have stressed the need for stricter oversight and public education about cosmetic products that contain corticosteroids. ^33^

### Limitations

This study design does not allow for the examination of long-term corticosteroid effects or the recovery of the hypothalamic-pituitary-adrenal (HPA) axis following cessation. Second, the sample size is sufficient for detecting statistical differences, but it may limit the generalizability of the findings to broader populations. Third, self-reported data on the quantity and duration of cream usage may introduce memory bias. They also did not take into account other things that could affect cortisol levels on their own, like stress, medication use, and food.

Furthermore, the variations in the formulation and potency of unregulated whitening products were not analyzed, which could influence the extent of systemic absorption. While an independent t-test was utilized to compare groups, additional statistical methods, such as multiple regression, could enhance the management of potential confounders and facilitate a more thorough interpretation of the results. In addition, ACTH levels were not measured, and dynamic endocrine testing was not performed; therefore, HPA-axis suppression could not be definitively confirmed. Considering the endocrine sensitivity exhibited by both genders, subsequent research should investigate whether sex-specific variations in skin permeability, corticosteroid metabolism, or hormonal fluctuations may influence systemic effects. To determine the causal relationships between topical corticosteroid application and adrenal suppression, subsequent research should emphasize longitudinal and interventional studies. To gain insights into the systemic effects and recovery duration of the HPA axis, biochemical analyses of the product’s composition and dose-response evaluations are advisable.

## Conclusions

The study's results demonstrate a significant association between the habitual use of corticosteroid-containing skin-whitening products and lower morning serum cortisol levels, which may suggest reduced HPA-axis activity. However, definitive confirmation of HPA-axis suppression requires additional endocrine evaluation, including ACTH measurement and dynamic hormonal testing. This study stresses how important it is to teach customers about the dangers that cosmetics can pose to the endocrine system.

Stricter laws governing the labeling and sale of skin-whitening products should be put into effect. Topical corticosteroids should only be used under a doctor’s care for diagnosed skin conditions, not for cosmetic whitening.

To more accurately describe the long-term effects of these practices on adrenal health, future research involving larger cohorts, hormonal panels (including ACTH), and longitudinal follow-up is advised.

## Ethical considerations

The study protocol was approved by the Institutional Review Board of the Ethical Approval Committee, Center of Technical Research, Northern Technical University, Iraq (Approval No: 11, Date: 2 October 2024).

## Data Availability

Figshare. The Effect of Whitening Creams Usage on the Adrenal Gland. https://doi.org/10.6084/m9.figshare.30840839. ^34^ The project contains the following underlying data:
•
BMI_Comparison_between_Control_and_Experimental_Groups Table 4.xlsx (this file contains calculated BMI values derived from the original height and weight data available in the same figshare repository which processed/summarized data rather than raw measurements.) • Control group information (cortisol levels, age and gender).xlsx (raw cortisol levels, age, and gender data for the control group) •
Cortisol_Below5_and_Below3 in users Figure 2.xlsx (raw cortisol suppression data used to generate Figure 2) •
Cortisol_Comparison_by_Gender_in_Experimental_Group Table 3.xlsx (this file contains calculated cortisol comparison values derived from the original cortisol datasets available within the same figshare repository. It represents processed/summarized data rather than raw participant level measurements) • Experimental group information (cortisol levels, age and gender).xlsx (raw cortisol levels, age, and gender data for the experimental group) •
Height_Weight_BMI_Table control group.xlsx (raw height, weight, and BMI data for the control group) •
Height_Weight_BMI_Table experimental group.xlsx (raw height, weight, and BMI data for the experimental group) Figshare. The Effect of Whitening Creams Usage on the Adrenal Gland. https://doi.org/10.6084/m9.figshare.30840839. ^34^ The project contains the following extended data:
•
Participant_Form_Whitening_Cream_Study Questionnaire. docx (Questionnaire used to collect participant information) • STROBE-checklist-v4-cross-sectional-whitening creams. doc (completed STROBE checklist for cross sectional study) • Ethical Approval Whitening Creams. jpg (Ethical Approval document for the study) The Data are available under the terms of the Creative Commons Zero “No rights reserved” data waiver (CC0 1.0 Public domain dedication). This study complies with the relevant reporting guidelines for observational human studies (STROBE).
