# Supplementary material for: The Effect of Whitening Creams Usage on the Adrenal Gland 
Source: F1000Res. 2026 Jul 31;14:1434. Originally published 2025 Dec 23. [Version 2] doi: 10.12688/f1000research.173725.2 (PMC13560007; doi:10.12688/f1000research.173725.2)
Supplement: Peer review history

# Reviewer report: Reviewer response for version 2

**DOI:** 10.5256/f1000research.206751.r506671

**Published:** 2026-08-03

**Authors:** Andrzej Slominski

**Affiliations:** 1 University of Alabama at Birmingham, Birmingham, AL, USA

The authors were responsive to critique and adequately revised the manuscript

Is the work clearly and accurately presented and does it cite the current literature?

Partly

If applicable, is the statistical analysis and its interpretation appropriate?

Yes

Are all the source data underlying the results available to ensure full reproducibility?

No source data required

Is the study design appropriate and is the work technically sound?

Yes

Are the conclusions drawn adequately supported by the results?

Partly

Are sufficient details of methods and analysis provided to allow replication by others?

Yes

Reviewer Expertise:

NA

I confirm that I have read this submission and believe that I have an appropriate level of expertise to confirm that it is of an acceptable scientific standard.

# Reviewer report: Reviewer response for version 1

**DOI:** 10.5256/f1000research.191564.r467715

**Published:** 2026-07-17

**Authors:** Andrzej Slominski

**Affiliations:** 1 University of Alabama at Birmingham, Birmingham, AL, USA

This manuscript addresses an important public health issue, namely the potential systemic effects of prolonged use of corticosteroid-containing skin-whitening products. The finding of lower morning serum cortisol levels among users is potentially relevant and consistent with previous reports of hypothalamic-pituitary-adrenal (HPA) axis suppression following chronic topical corticosteroid exposure. However, the study has several conceptual and methodological weaknesses that limit its impact.

 Major Concerns

1. **Insufficient understanding of skin endocrinology: **
  The major intellectual weakness of the manuscript is its presentation of the skin as merely a passive route for corticosteroid absorption. The authors discuss only the central HPA axis and adrenal function while completely ignoring the well-established concept that the skin possesses its own **neuro-immuno-endocrine system**, including a functional **cutaneous HPA-axis equivalent (**Neuro–immuno–endocrinology of the skin: how environment regulates body homeostasis. *Nat Rev Endocrinol* (2025), Vol. 21 Issue 8 Pages 495-509. https://doi.org/10.1038/s41574-025-01107-x ) and local steroidogenic machinery (Mol Cell Endocrinol. 2021 Jun 15;530:111238. PMID: 33716049). Human skin produces CRH, POMC peptides, ACTH, cortisol, and related mediators and functions as an independent peripheral neuroendocrine organ. Failure to consider this extensive literature results in an oversimplified and outdated biological framework.
2. **Overinterpretation of cortisol measurement:** 
  The conclusion that reduced serum cortisol demonstrates “adrenal suppression” is not sufficiently supported. A single morning cortisol measurement cannot establish HPA-axis suppression or secondary adrenal insufficiency. Confirmation would require ACTH measurements and ideally dynamic testing (e.g., ACTH stimulation test). Therefore, the conclusions should be substantially tempered.
3. **Lack of characterization of whitening products**:
  The products were described as cosmetic mixtures obtained from beauty salons, yet no chemical analysis was performed. Consequently, the actual corticosteroid content, potency, dose, and exposure cannot be determined. This severely limits causal inference.
4. **Small sample size and cross-sectional design**:
  The study is observational and cross-sectional, preventing conclusions regarding causality. The modest sample size and convenience sampling further limit generalizability.
**Minor Concerns**
  The discussion contains numerous grammatical and linguistic errors that require substantial editing.
  Several references are only tangentially related to the study objectives.
  Potential confounders affecting cortisol levels (stress, sleep, medication use, menstrual phase, smoking, comorbidities) were not adequately controlled.
  Statistical analyses are simplistic and do not account for potential covariates

Is the work clearly and accurately presented and does it cite the current literature?

Partly

If applicable, is the statistical analysis and its interpretation appropriate?

Yes

Are all the source data underlying the results available to ensure full reproducibility?

No source data required

Is the study design appropriate and is the work technically sound?

Yes

Are the conclusions drawn adequately supported by the results?

Partly

Are sufficient details of methods and analysis provided to allow replication by others?

Yes

Reviewer Expertise:

NA

I confirm that I have read this submission and believe that I have an appropriate level of expertise to confirm that it is of an acceptable scientific standard, however I have significant reservations, as outlined above.

# Response

**Published:** 2026-07-20

**Authors:** نور فيصل نعمان عباده

**Thank you very much for your valuable and constructive review. We sincerely appreciate your insightful comments and suggestions. We have carefully revised the manuscript in response to all of your comments, and a revised Version 2 has now been submitted. The revised manuscript includes an updated Introduction incorporating recent literature on cutaneous neuroendocrinology, a more cautious interpretation of the cortisol findings, clarification of the study limitations, and discussion of the lack of chemical characterization of the whitening products. We sincerely thank you for your thoughtful review, which has helped us improve the quality and scientific rigor of our manuscript.**

# Reviewer report: Reviewer response for version 1

**DOI:** 10.5256/f1000research.191564.r446456

**Published:** 2026-01-29

**Authors:** Widyastuti Widyastuti

**Affiliations:** 1 Universitas Perintis Indonesia, Padang, West Sumatra, Indonesia

This study utilized current literature. Because it consisted of two groups, statistical analysis using the t-Student test was representative. The reproducibility of the method can be carried out by other researchers. The research results are essential as information for whitening product users, encouraging them to be more selective in choosing whitening products.

Is the work clearly and accurately presented and does it cite the current literature?

Yes

If applicable, is the statistical analysis and its interpretation appropriate?

Yes

Are all the source data underlying the results available to ensure full reproducibility?

Yes

Is the study design appropriate and is the work technically sound?

Yes

Are the conclusions drawn adequately supported by the results?

Yes

Are sufficient details of methods and analysis provided to allow replication by others?

Yes

Reviewer Expertise:

Sunscreen, skin lightening, and antiaging from natural products

I confirm that I have read this submission and believe that I have an appropriate level of expertise to confirm that it is of an acceptable scientific standard.
